# Implementation of a One-Day Living Kidney Donor Assessment Clinic to Improve the Efficiency of the Living Kidney Donor Evaluation: Program Report

**DOI:** 10.1177/20543581241231462

**Published:** 2024-02-25

**Authors:** Seychelle Yohanna, Kyla L. Naylor, Jessica M. Sontrop, Christine M. Ribic, Catherine M. Clase, Matthew C. Miller, Sunchit Madan, Richard Hae, Jasper Ho, Jian Roushani, Sarah Parfeniuk, Melodie Jansen, Sharon Shavel, Michelle Richter, Kimberly Young, Brooke Cowell, Shahid Lambe, Peter Margetts, Kevin Piercey, Vikas Tandon, Colm Boylan, Carol Wang, Susan McKenzie, Barb Longo, Amit X. Garg

**Affiliations:** 1 Department of Medicine, McMaster University, Hamilton, ON, Canada; 2 St. Joseph’s Healthcare Hamilton, ON, Canada; 3 Department of Epidemiology and Biostatistics, Western University, London, ON, Canada; 4 Lawson Health Research Institute, London Health Sciences Centre, ON, Canada; 5 Canadian Blood Services, Ottawa, ON, Canada; 6 Division of Urology, Department of Surgery, McMaster University, Hamilton, ON, Canada; 7 Division of Cardiology, McMaster University, Hamilton, ON, Canada; 8 Department of Radiology, McMaster University, Hamilton, ON, Canada; 9 Department of Medicine, Western University, London, ON, Canada; 10 Transplant Ambassador Program, Toronto, ON, Canada

**Keywords:** living kidney donation, donor evaluation, kidney transplant, clinic

## Abstract

**Purpose of program:**

A key barrier to becoming a living kidney donor is an inefficient evaluation process, requiring more than 30 tests (eg, laboratory and diagnostic tests), questionnaires, and specialist consultations. Donor candidates make several trips to hospitals and clinics, and often spend months waiting for appointments and test results. The median evaluation time for a donor candidate in Ontario, Canada, is nearly 1 year. Longer wait times are associated with poorer outcomes for the kidney transplant recipient and higher health care costs. A shorter, more efficient donor evaluation process may help more patients with kidney failure receive a transplant, including a pre-emptive kidney transplant (ie, avoiding the need for dialysis). In this report, we describe the development of a quality improvement intervention to improve the efficiency, effectiveness, and patient-centeredness of the donor candidate evaluation process. We developed a One-Day Living Kidney Donor Assessment Clinic, a condensed clinic where interested donor candidates complete all testing and consultations within 1 day.

**Sources of information:**

The One-Day Living Kidney Donor Assessment Clinic was developed after performing a comprehensive review of the literature, receiving feedback from patients who have successfully donated, and meetings with transplant program leadership from St. Joseph’s Healthcare Hamilton. A multistakeholder team was formed that included health care staff from nephrology, transplant surgery, radiology, cardiology, social work, nuclear medicine, and patients with the prior lived experience of kidney donation. In the planning stages, the team met regularly to determine the objectives of the clinic, criteria for participation, clinic schedule, patient flow, and clinic metrics.

**Methods:**

Donor candidates entered the One-Day Clinic if they completed initial laboratory testing and agreed to an expedited process. If additional testing was required, it was completed on a different day. Donor candidates were reviewed by the nephrologist, transplant surgeon, and donor coordinator approximately 2 weeks after the clinic for final approval. The team continues to meet regularly to review donor feedback, discuss challenges, and brainstorm solutions.

**Key findings:**

The One-Day Clinic was implemented in March 2019, and has now been running for 4 years, making iterative improvements through continuous patient and provider feedback. To date, we have evaluated more than 150 donor candidates in this clinic. Feedback from donors has been uniformly positive (98% of donors stated they were very satisfied with the clinic), with most noting that the clinic was efficient and minimally impacted work and family obligations. Hospital leadership, including the health care professionals from each participating department, continue to show support and collaborate to create a seamless experience for donor candidates attending the One-Day Clinic.

**Limitations:**

Clinic spots are limited, meaning some interested donor candidates may not be able to enter a One-Day Clinic the same month they come forward.

**Implications:**

This patient-centered quality improvement intervention is designed to improve the efficiency and experience of the living kidney donor evaluation, result in better outcomes for kidney transplant recipients, and potentially increase living donation. Our next step is to conduct a formal evaluation of the clinic, measuring qualitative feedback from health care professionals working in the clinic and donor candidates attending the clinic, and measuring key process and outcome measures in donor candidates who completed the one-day assessment compared with those who underwent the usual care assessment. This program evaluation will provide reliable, regionally relevant evidence that will inform transplant centers across the country as they consider incorporating a similar one-day assessment model.

## Abrégé

**Objectifs du programme:**

Devenir donneur de rein vivant est difficile, le principal obstacle étant le processus d’évaluation inefficace auquel les candidats doivent se soumettre. Ce processus comporte plus de 30 examens (p. ex. tests de laboratoire et tests diagnostiques), questionnaires et consultations avec des spécialistes. Les candidats donneurs font plusieurs visites dans les hôpitaux et cliniques, et passent souvent plusieurs mois à attendre des rendez-vous et des résultats de tests. En Ontario (Canada), le délai médian pour l’évaluation d’un candidat au don est de près d’un an. Les temps d’attente plus longs sont associés à de moins bons résultats pour les receveurs d’une greffe rénale, ainsi qu’à des coûts de soins de santé plus élevés. Un processus d’évaluation plus court et plus efficace des donneurs potentiels permettrait à un plus grand nombre de patients atteints d’insuffisance rénale de recevoir une greffe, y compris une greffe préventive (c.-à-d. permettant d’éviter la dialyse). Cet article décrit une intervention d’amélioration de la qualité visant à augmenter l’efficience, l’efficacité et la personnalisation du processus d’évaluation des candidats au don. Nous avons développé une clinique d’un jour pour l’évaluation des donneurs de reins vivants (One-Day Living Kidney Donor Assessment Clinic), soit une clinique condensée où les candidats passent tous les tests et consultent un spécialiste dans la même journée.

**Sources de l’information:**

La clinique d’un jour pour l’évaluation des donneurs de reins vivants a été développée à la suite d’un examen approfondi de la littérature, de la consultation des commentaires de patients ayant donné avec succès et de rencontres avec les dirigeants du programme de transplantation du St Joseph’s Healthcare d’Hamilton. Une équipe multipartite a été formée; celle-ci réunit du personnel soignant en néphrologie, chirurgie de transplantation, radiologie, cardiologie, travail social et médecine nucléaire, ainsi que des patients ayant une expérience vécue du don de rein. L’équipe s’est réunie régulièrement pendant les étapes de planification pour déterminer les objectifs, les paramètres et le calendrier de la clinique, ainsi que les critères de participation et le flux de patients.

**Méthodologie:**

Les donneurs potentiels qui avaient complété les tests de laboratoire initiaux et qui acceptaient de se soumettre à un processus accéléré ont été évalués à la clinique d’un jour. Si des tests supplémentaires étaient nécessaires, ceux-ci étaient effectués un autre jour. Les candidats ont été rencontrés par le néphrologue, le chirurgien de transplantation et le coordonnateur des dons environ deux semaines après leur visite à la clinique pour l’approbation finale. L’équipe multipartite continue de se réunir régulièrement pour examiner les commentaires des donneurs, discuter des défis et trouver des solutions.

**Principaux résultats:**

La clinique d’un jour, mise sur pied en mars 2019, est en activité depuis quatre ans et permet des améliorations itératives grâce à la rétroaction continue des patients et des soignants. À ce jour, plus de 150 candidats au don ont été évalués à la clinique. Les commentaires des donneurs sont quasi unanimement positifs (98 % des candidats ont déclaré être très satisfaits de la clinique), la plupart soulignant l’efficacité de la clinique et les conséquences minimes du processus sur les obligations professionnelles et familiales. La direction de l’hôpital, tout comme les professionnels de la santé des services participants, continue d’appuyer la clinique d’un jour et de collaborer à la création d’une expérience fluide pour les donneurs potentiels qui la fréquentent.

**Limites:**

Les places à la clinique sont limitées; ainsi, certains candidats au don d’un rein vivant pourraient ne pas pouvoir être admis dans le mois où ils se présentent à la clinique.

**Conclusion:**

Cette intervention d’amélioration de la qualité axée sur les patients est conçue pour augmenter l’efficacité du processus d’évaluation et bonifier l’expérience des donneurs de rein vivants. Elle vise également à améliorer les résultats des receveurs d’une greffe rénale et, potentiellement, augmenter le don vivant. La prochaine étape sera une évaluation formelle de la clinique, c’est-à-dire la mesure de la rétroaction qualitative des professionnels de la santé qui y travaillent et des candidats au don qui la fréquentent, et l’analyse des processus clés et des résultats des candidats évalués à la clinique d’un jour par rapport à ceux qui suivent le processus d’évaluation habituel. Cette évaluation du programme fournira des données probantes fiables et propres à la région qui pourront informer les centres de transplantation de tout le pays qui envisagent d’intégrer un processus d’évaluation similaire.

## Introduction

Kidney transplantation offers patients with kidney failure a longer and better quality of life at a fraction of the cost of dialysis.^1^ Every 100 kidney transplants in Canada saves the health care system at least $20 million in avoided dialysis costs.^2,3^ However, the number of potential recipients is greater than the number of living and deceased donors, leading to delays in transplantation, and some potentially eligible patients becoming too sick to receive a transplant or dying before transplantation.^4^ In Canada, living donor kidney transplant rates have not risen since 2006.^4-6^

The evaluation process to become a living kidney donor is lengthy, inefficient, and has been identified as a significant barrier to donation.^7^ A donor candidate must complete an extensive health-related questionnaire, numerous clinical tests, receive education, and attend many medical, surgical, and social work consultations. These appointments are often on different days and in different locations. A prolonged donor evaluation affects both the donor candidate and their intended recipient. First, there are financial concerns for donors who must take time away from work and other responsibilities.^8^ Second, donors experience anxiety and confusion navigating the pathway to donation.^9-11^ Williams et al^9^ found that when donors experience a long period of evaluation, they describe a “weight” to bear and a feeling that they “lack control.” Donors become increasingly concerned that their intended recipient’s health will deteriorate and worry about the impact of the recipient’s illness on their quality of life.^10^ Finally, a lengthy or delayed donor evaluation often results in the intended recipient having to start dialysis while waiting for their donor to be medically cleared.^12^ In Ontario, 26% of recipients who had an identified living donor in the year prior to transplant started dialysis urgently in hospital resulting in $8 million in dialysis costs to the health care system.^13^ This is also an important failing because pre-emptive transplantation (ie, transplant prior to receiving dialysis) offers improved survival and is the most cost-effective treatment for patients with kidney failure.^14,15^ The length of the donor assessment is a quality metric used in some Canadian transplant programs.^16^ In this report, we describe the development, implementation, and early lessons learned of the first One-Day Living Kidney Donor Assessment Clinic launched in Ontario, Canada. This is a condensed clinic where donor candidates complete all testing and consultations within 1 day.

### Examining Our Local Process

**Figure 1.**
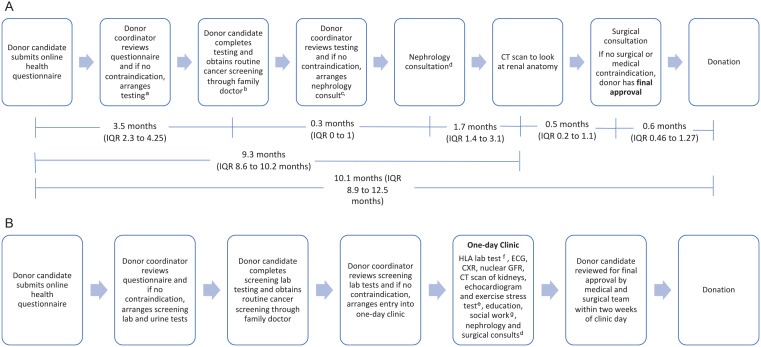
(A) Usual Care Donor evaluation process, including the time to complete each major milestone. (B) One-day donor evaluation process. *Note.* CT = computed tomography; IQR = interquartile range; GFR = glomerular filtration rate; HLA = human leukocyte antigen, ECG = electrocardiogram, CXR = chest x-ray. ^a^Blood and urine lab tests, nuclear GFR, chest x-ray, electrocardiogram, echocardiogran and exercise stress testing in candidates greater than 50 years old or with prior cardiac concerns. Tests usually scheduled on multiple days. ^b^HLA testing and flow crossmatch are ordered at varying times depending on clinical circumstance. ^c^Social work consultation occurs virtually prior to nephrologist consultation. ^d^Other testing may be required depending on donor’s medical history or test results. ^e^HLA lab test is drawn and stored on clinic day. Flow crossmatch is completed subsequently if donor deemed eligible. ^f^Echocardiogram and exercise stress testing in candidates greater than 50 years old or with prior cardiac concerns. ^g^Depending on availability, social work consultation occurs either in person during clinic or virtually prior to clinic day.

Figure 1A outlines the usual care donor evaluation process at the St. Joseph’s Healthcare Hamilton Living Donor program (Hamilton, Ontario, Canada), including median evaluation times measured for donor candidates beginning the assessment from January 1, 2016, to December 31, 2018. Overall, the median time from a donor candidate first contacting our transplant center to donation was 10.1 months (interquartile range [IQR], 8.9-12.5 months). This is consistent with Canadian and Australian transplant centers reported by Habbous et al,^12^ for which the median time from contacting a transplant center to completing the assessment and receiving surgery was 10.3 months (IQR, 6.5-17 months). Donor candidates in our program typically make 9 to 15 trips to the hospital to complete the assessment.

Canada lacks a national benchmark for the length of the donor evaluation, and the Canadian commentary on the KDIGO (Kidney Disease: Improving Global Outcomes) guidelines for evaluation of living donor candidates provides no guidance on process issues or timelines.^17^ We believe that the evaluation for a motivated donor candidate without added complexities (such as the need for weight loss or a native kidney biopsy) should ideally be completed within 3 to 6 months (where the candidate is given an answer whether they are eligible to donate or not). A high-level research priority identified by the American Society of Transplantation is for transplant programs to use quality improvement initiatives to optimize the donor evaluation process. The aim is to remove inefficiencies and provide timely decisions on whether a donor is accepted or rejected.^15,18,19^

In 2016, we sent a survey to all living kidney donors who donated a kidney from 2012 to 2016, 3 months after donation that focused on their experience during the evaluation process (n = 44, 29% response rate). Thirty-four percent of donors stated the evaluation process took too long to complete. Feedback included statements like “speed up the process,” “long time from start to finish,” and “more appointments on one day.” One donor stated, “I found out when I submitted parking receipts for reimbursement that I visited the hospital 15 times.” In narrative accounts of donor experiences, Laneuville et al^20^ reported a donor writing, “The fear of rejection is always there, isn’t it?” and another “At times, I found myself wondering: am I still fit to donate a kidney? Will they find something else I don’t know about? A cancer somewhere?” To improve the living donor evaluation process at our center, we established a team comprised of key stakeholders with the aim of creating a more efficient and streamlined process. Team members included living kidney donors who had successfully donated a kidney, health care staff from the Living Donor program (ie, nurse coordinators, administrative staff, clinic manager, and physicians), nuclear medicine, radiology, transplant surgery, cardiology, and social work. The team discussed barriers to living kidney donation and decided that the most efficient and patient-centered way to evaluate living donors would be to have them attend a One-Day Clinic where donor candidates would complete all standardized testing and consultations in 1 day.

### St. Joseph’s Healthcare Hamilton Living Donor Program

The St. Joseph’s Healthcare Hamilton kidney transplant program is 1 of 6 adult transplant centers in Ontario. In 2022, we performed 126 total transplants (37 living donor kidney transplants), making St. Joseph’s Healthcare the third largest transplant program in Ontario. Five of 27 chronic kidney disease (CKD) programs in the province refer patients for transplant assessment to Hamilton, one of which is the local Hamilton CKD program. Our transplant program has 2 full-time donor coordinators, 2 full-time recipient coordinators, 1 part-time recipient coordinator, 5 nephrologists who see potential transplant recipients, 3 nephrologists who see living donor candidates, and 2 administrative support staff. Our program policy is to complete initial laboratory testing for up to 5 donor candidates and the full evaluation for 2 donor candidates simultaneously for each recipient, unless a recipient is pre-emptive, in which case a discussion occurs between the donor coordinator and nephrologist to determine whether additional donor candidates should be evaluated. Our program has a psychologist who sees donor candidates with any history of mental illness, solicited donors, anonymous donor candidates (directed or non-directed), or any donor candidate thought to benefit from additional psychological evaluation. The donor team meets weekly to review newly submitted donor health questionnaires with questionable health history or donor candidates in evaluation where questions of eligibility have been raised. Our evaluation and acceptance criteria follow what is described in the Kidney Paired Donation Protocol for Participating Donors, developed by the Canadian Blood Services Living Donor Advisory Committee.^21^ This protocol outlines acceptance criteria, absolute contraindications, and potential exclusions for donor candidates participating in the kidney paired exchange program, and its purpose is to promote coordination of assessment and acceptance practices across Canada. Three transplant surgeons perform approximately one living donor transplant each week (additional transplant dates can be requested based on program needs). At bimonthly surgical meetings, we review surgical issues for donors and recipients and at weekly transplant program meetings we review recipients, upcoming living donor kidney transplants and pairs being entered into the Kidney Paired Donation program.^22,23^ Patient peers provide a unique and important perspective for donor candidates undergoing the evaluation. We suggest that donor candidates connect with a transplant ambassador (a patient peer with the lived experience of kidney donation; https://transplantambassadors.ca/) at the time of health questionnaire submission (a box can be checked to indicate their interest in speaking with an ambassador), during their education session with the donor coordinator, and at their social work visit.

## Methods

### Development and Implementation of the One-Day Clinic

The Hamilton Integrated Research Ethics Board deemed this project to be a quality improvement activity (Tri-Council Policy Statement: Ethical Conduct for Research Involving Humans—TCPS 2 Article 2.5) and as a result granted an exemption from an ethics review.

#### Phase 1: Donor candidate screening process

We developed a 6-person working group that focused on improving the efficiency of the screening process. This group consisted of nephrologists, donor coordinators, administrative staff, and the clinic manager. We included frontline staff in the project team from the beginning to increase support for change. Our initial work aimed at ensuring our screening process for all donor candidates was optimized and that our testing requirements were evidence based and aligned with the Kidney Paired Donation Protocol for Participating Donors.^21^ We conducted an audit of all donor candidates who contacted our center in 2016 (n = 246) and spent time shadowing donor coordinators to observe workflow. We found that donor coordinators were spending up to 30 minutes with each donor candidate on the telephone asking health screening questions and answering questions about the donation and transplantation process. At the end of the telephone call, if there were no obvious health contraindications and the donor candidate agreed, they would mail out the health questionnaire. Our audit showed that only 50% of donor candidates returned the health questionnaire and only 48% of those went on to complete the evaluation. As we only have 2 donor coordinators, telephone screening was a significant time resource that was resulting in less time available to navigate remaining donors efficiently through the process. Furthermore, the use of regular mail to send and submit the health questionnaire was resulting in a screening process that was taking a median of 28 days. As a result, telephone intake and health screening by donor coordinators were eliminated and the process for donor candidates coming forward became electronic self-referral: completion of the health questionnaire linked to our program website became the first step in the process (https://www.stjoes.ca/living-kidney-donor-program). Each of our referring CKD programs was given printed cards with the health questionnaire website and donor program contact information. Donor candidates can also find the link to complete the questionnaire using their Internet browser and navigating to our living donor program website. As many of our donor candidates eventually enter the Kidney Paired Donation program, we used the same health questionnaire that is required by Canadian Blood Services. Required consent forms were also housed on our living donor program website so that donor candidates could easily access, complete, and submit forms online. After reviewing our testing requirements, we removed testing and consultations that did not align with evidence-based practice (eg, mandatory 24-hour ambulatory blood pressure monitoring and cardiology consultations in low-risk donor candidates). A list of our required tests and consultations can be found in Appendix A.

#### Phase 2: Meetings with program leadership and key stakeholders

To inform the need for system-level changes to the donor evaluation process, we reviewed the results of the donor survey and evaluation process audit with program leadership. To find potential process improvements, we conducted a literature review and found a 2018 report describing the creation of a 1-day clinic in Northern Ireland, which resulted in increased rates of living donor kidney transplantation from an annual rate of 4 per million population to 33 per million population.^24^ We presented the results of this study to the nephrology department to begin to socialize the idea of developing a local One-Day Clinic. We also engaged prior kidney donors who sat on our patient and family advisory committee to discuss the idea of a 1-day assessment, to obtain feedback about the design and create a plan for ongoing patient engagement. We eventually received support to design and implement a local 1-day assessment. This approval included pronouncing the initiative as a major theme and strategy of the nephrology program at our Nephrology Quality Advisory Committee meeting.

No new funding was assigned to the One-Day Clinic, which required resources from multiple hospital departments; organizational barriers have previously been identified as an issue for living donation.^25^ In Fall 2018, we reached out to administrative and medical leadership in nuclear medicine, cardiology, radiology, social work, and transplant surgery. During individual meetings with each group of stakeholders, we reviewed the benefits of receiving a living donor kidney transplant and described how the number of living donor kidney transplants we were performing each year had been stagnant, potentially due to an inefficient donor evaluation process. We presented local cases that demonstrated the current living donor evaluation experience at our center, highlighting how long it took donor candidates to complete the process and how many visits to hospital were required. For each stakeholder group, we outlined our vision for the One-Day Clinic, and what was needed from them to help realize the vision. For example, in discussion with radiology, we planned how we would identify donors on arrival, insert peripheral intravenous lines for contrast (that would need to be kept in place for the nuclear imaging renal function test completed later in the day), perform a standardized donor protocol computed tomography (CT) scan (multiphase acquisition of images post contrast to allow assessment of renal veins, parenchyma, and collecting systems), chest x-rays, and direct donors to their next testing location. We planned to alternate the days of the clinic based on transplant surgeon availability and agreed that we would select dates 3 months in advance to give departments the ability to schedule around other hospital priorities.

#### Phase 3: One-Day Clinic design

**Figure 2.**
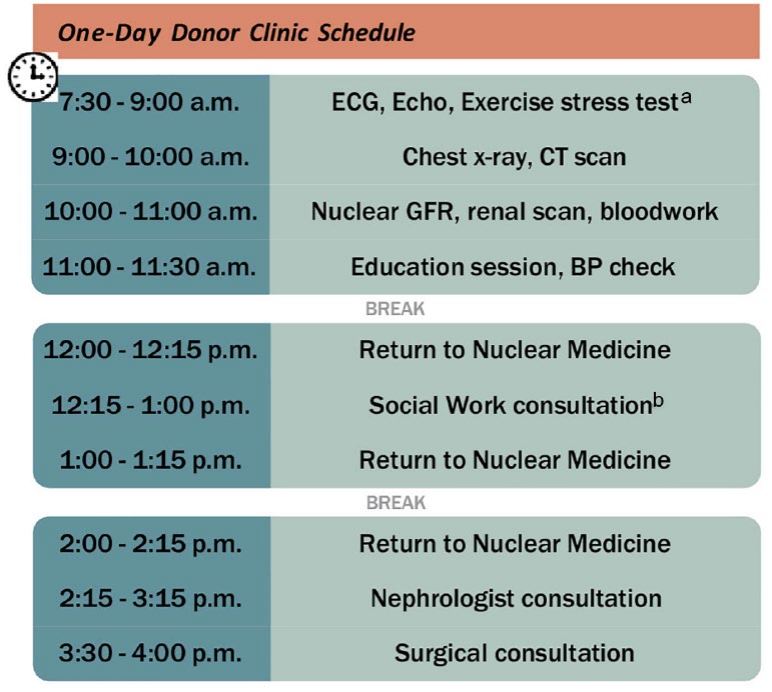
Example One-Day Clinic schedule. *Note.* ECG = electrocardiogram; CT = computed tomography; GFR = glomerular filtration rate; BP = blood pressure. ^a^Echo and stress test only done for patients ≥50 years old or in special cases where donor team feels it is necessary. ^b^Depending on availability, social work consultations occur in person during clinic or virtually prior to the clinic.

An example of the schedule for one of the 4 donor candidates in the clinic is shown in Figure 2. The tests included in the One-Day Clinic are the same as those required by donor candidates in a usual care pathway (Appendix A). We perform echocardiogram and exercise stress testing for donor candidates 50 years or older, or those with a risk factor (eg, hypertension, smoking) for cardiac disease. If abnormalities in testing are found which require further investigation, subsequent testing is scheduled on another day, and a decision regarding suitability would be delayed until testing is complete. Our initial criteria for participation in the One-Day Clinic were that the donor candidate wanted an expedited assessment and that initial basic laboratory testing had been completed and reviewed before the day. Donor candidates were 18 years of age or older (there is no upper age limit for participation). We evaluated only 1 or 2 donor candidates for the same recipient in a single clinic. Additional donor candidates for a recipient were evaluated at a subsequent One-Day Clinic or in the usual care pathway. Age-appropriate cancer screening was organized by the donor candidate’s family physician and therefore was not included in this day. We reviewed all donor candidates for final approval approximately 2 weeks later at a multidisciplinary meeting. All donor candidates (those who completed the One-Day assessment and those who completed the usual care assessment) were seen by the donor’s physician within 30 days of the planned surgery (as required by Health Canada) and by anesthesia and surgery 1 week before donation. The One-Day clinic donor evaluation process is shown in Figure 1B.

#### Phase 4: Implementation of the One-Day Clinic

We held the first One-Day Clinic on March 5, 2019, and continued monthly clinics, evaluating 4 donor candidates per clinic day (which is approximately 25% of assessments scheduled each month). For the first few clinics, hospital volunteers helped donors to navigate around the hospital to ensure the flow of the clinic was maintained. We administered a survey at the end of each clinic requesting feedback from all donor candidates to iteratively improve the implementation of the One-Day Clinic (Appendix B). For example, in the beginning, we had donor candidates go to the hospital’s outpatient laboratory for their human leukocyte antigen (HLA) blood test, which was a 5-minute walk from the rest of the testing locations; donors indicated that this distance made it difficult to make it to their next appointment on time. As a result, we worked with our nuclear medicine team to include the HLA blood test in the bloodwork at the same time as collecting blood for the nuclear glomerular filtration rate (GFR) and renal scan. We had quarterly meetings with the larger hospital group to share information, discuss any issues that arose, and troubleshoot solutions as a team. Transplant ambassadors attended the clinic in person to offer support for donors during their breaks (this was paused only during the most critical waves of the COVID-19 pandemic). Before the pandemic, the social work visit was integrated into the clinic schedule; however, since June 2020, social work visits have been virtual (completed in advance of the One-Day Clinic). While in-person social work visits are now permitted, keeping them virtual allowed for the additional break time during the One-Day Clinic that donors were asking for.

### Key Findings

We monitored several clinic metrics and reported them quarterly at Living Donor Transplant Committee meetings held at our hospital. For example, we reported the number of donor candidates participating in the clinic and final disposition of donor candidates participating in the program (eg, number of donor candidates approved, reasons for ineligibility, proportion of donor candidates who have donated). From March 2019 to November 2023, 202 donor candidates participated in the One-Day Clinic. We administered a post-clinic survey (Appendix B), which was completed in person during their last appointment and collected by our administrative staff. Results of this survey (n = 42) indicated that 95% of donor candidates strongly agreed the clinic ran smoothly, 90% strongly agreed that they received the information they needed to make a decision about kidney donation, and 95% were very satisfied with their experience during the clinic. We reviewed feedback at our team meetings and adjusted the day as needed.

### Lessons Learned

As we reflect on the One-Day Clinic over the past 3 years, a key strength of the clinic is the teamwork and generosity of different departments that were necessary for initiating and maintaining this initiative. Every member of the team exhibits pride and ownership of the clinic’s success, and this success has been highlighted at our hospital’s board meetings, at the hospital’s Advancing Patient, Family and Staff Partnerships event “My voice matters,” by the hospital’s social media account and by Cancer Care Ontario (www.thespec.com). The strong teamwork developed by engaging stakeholders early in the development, and repeatedly engaging them over time, allows them to feel integral to the clinic’s implementation. Donor candidates greatly appreciated the opportunity to participate in the clinic. Even when donor candidates are deemed ineligible, they appreciated knowing early on so they could help their recipient identify other potential donors. Soliciting donor feedback and incorporating changes to address their concerns demonstrates our commitment to patient engagement and ensuring our process is patient-centered. Table 1 shows the main modifications made to the clinic based on donor candidate and health care provider feedback obtained during the early implementation of the clinic.

**Table 1.** Main Modifications Made to the Clinic Based on Donor Candidate and Health Care Provider Feedback.

Adjust start times earlier so that donors have enough time to complete tests at each testing location
Have donors wear brightly colored lanyards so they are recognized by health care staff when presenting at testing locations across the hospital
Have transplant ambassadors attend the clinic to help orient donors and be available to provide the lived experience of living donation
Include human leukocyte antigen (HLA) blood work with the bloodwork taken for the nuclear medicine imaging scan
Provide clear instructions about what food and/liquids can be taken on the day
Provide a better hospital map that shows exactly where the testing locations are

At the beginning, we aimed to determine a candidate’s eligibility by the end of the One-Day Clinic. Unfortunately, this was not possible as the final reports for diagnostics tests (eg, CT scan of the abdomen, echocardiogram, and nuclear GFR and renal scan) were not available within a few hours of the test. For example, subspecialist abdominal radiologists were not able to accommodate urgent CT readings among other hospital priorities, and the team agreed that donor candidate safety was a priority and that rushed readings or non-specialist radiologist readings might lead to errors. We learned early on that when a donor candidate was late or was lost finding their next appointment, it set off a series of downstream scheduling issues throughout the day. To remedy this, we altered the schedule to ensure donors arrived earlier, and we gave them brightly colored lanyards to help staff in each department identify the One-Day donor candidates and prioritize their testing. We also revised the hospital map provided to donor candidates several times based on where we found they were most frequently getting lost. Because clinic spots are a limited resource, the donor coordinators used their judgment when selecting candidates, trying to maximize the benefit that could be achieved with an expedited assessment. For example, we found that some donors presented to clinic with a body mass index higher than reported on their health questionnaire, and if higher than 35 kg/m^2^, these donors would be ineligible or have a delayed time to approval. As a result, we modified the entry criteria such that any donor with a body mass index at or above the eligibility cutoff of 35 kg/m^2^ (set by the KPD program) would not meet the criteria for entry into the One-Day Clinic. Although one of the goals of this clinic was to increase pre-emptive transplants, when we evaluated candidates whose recipients had eGFRs >15 mL/min/1.73 m^2^, we found that donor candidates were ready far in advance of their intended recipient’s readiness for transplant, which meant the donor’s testing could become outdated. For this reason, we subsequently changed clinic eligibility to candidates whose intended recipient had an eGFR ≤15 mL/min/1.73 m^2^. Finally, we agreed to evaluate up to 2 candidates for one intended recipient during the same One-Day Clinic if the recipient was pre-emptive and progressing rapidly to kidney failure. In most cases, we evaluated multiple donor candidates for a recipient in sequential One-Day Clinics or allowed additional donor candidates to complete the evaluation in the usual care pathway. These decisions were made on a case-by-case basis by the donor coordinators and nephrologists.

## Discussion

The standard living kidney donor evaluation process is not patient-centered; it takes too long to complete and requires too many trips to the hospital. In this article, we describe the planning, implementation, and early lessons learned from the One-day Living Kidney Donor Assessment Clinic at St. Joseph’s Healthcare Hamilton in Ontario. To our knowledge, this is the first clinic of its kind to launch in Canada. We anticipate that this initiative will improve the efficiency and patient-centeredness of the living kidney donor evaluation process and potentially increase the number of living donor kidney transplants. Development of this clinic required multistakeholder engagement with nephrology, transplant surgery, radiology, cardiology, nuclear medicine, and social work. We successfully launched the clinic in March 2019 and evaluated 202 donor candidates as of November 2023.

A one-day clinic has been previously described in the literature. In 2017, Graham et al published their results from implementation of a 1-day evaluation clinic in Northern Ireland. Over a 5-year period (2010-2015), 431 donors participated in the 1-day evaluation, 66% of whom ultimately donated. The study reported an increase in the annual rate of living donor kidney transplantation from a mean of 4.3 per million population from 2000 to 2009 to 32.6 per million population from 2011 to 2015. In addition, there was an increase in the annual proportion of pre-emptive living donor kidney transplants from <10% before 2010 to >50% in 2015. There are a few key differences between our 1-day evaluation and the one described by Graham et al. First, the criteria for entry are different. Entry into the one-day clinic in Northern Ireland was possible upon completion of the health questionnaire, while we complete basic laboratory testing before the 1-day evaluation. Because we have only 4 spots each month, we maximized their utility by excluding anyone likely to be ineligible based on routine laboratory testing (eg, low eGFR or elevated HbA1C). Second, we included both cardiac testing (for donor candidates older than 50 years) and surgical consultation, whereas the Northern Ireland 1-day evaluation did not.

There are limitations to the design and implementation of our clinic. First, only 4 donor candidates could be assessed in the clinic each month, meaning some candidates who wanted to participate in the One-Day Clinic had to wait until later months or complete their evaluation in the usual care manner. Second, the COVID-19 pandemic led to delays in testing, completing recipient assessments, and living donor transplant surgeries, potentially reducing the impact of the expedited donor evaluation. Third, the improvements to the screening process described in Phase 1 did not include a patient partner.

Facilitating an efficient and patient-centered donor evaluation is of paramount importance to improving the experience of donation and ultimately increasing living donor kidney transplantation. Reducing the donor assessment time by 3 months has been estimated to save the Canadian health care system $12 055 per recipient and to result in ~26% more kidney transplants per year.^26^ This article provides a comprehensive description of the development of this quality improvement intervention, the team involved in the work, and our local context in which the clinic was implemented. Our next step will be to complete a formal qualitative and quantitative evaluation of the clinic. We will assess donor and recipient process, balancing and outcome measures and examine the impact of the 1-day assessment clinic on outcomes for transplant recipients (eg, number of pre-emptive transplants). If we demonstrate improvements in process and outcome measures, we will use our findings to inform and, we hope, inspire other transplant centers in Canada and beyond to adopt a similar model of donor assessment.

## Figures and Tables

**Appendix B.**
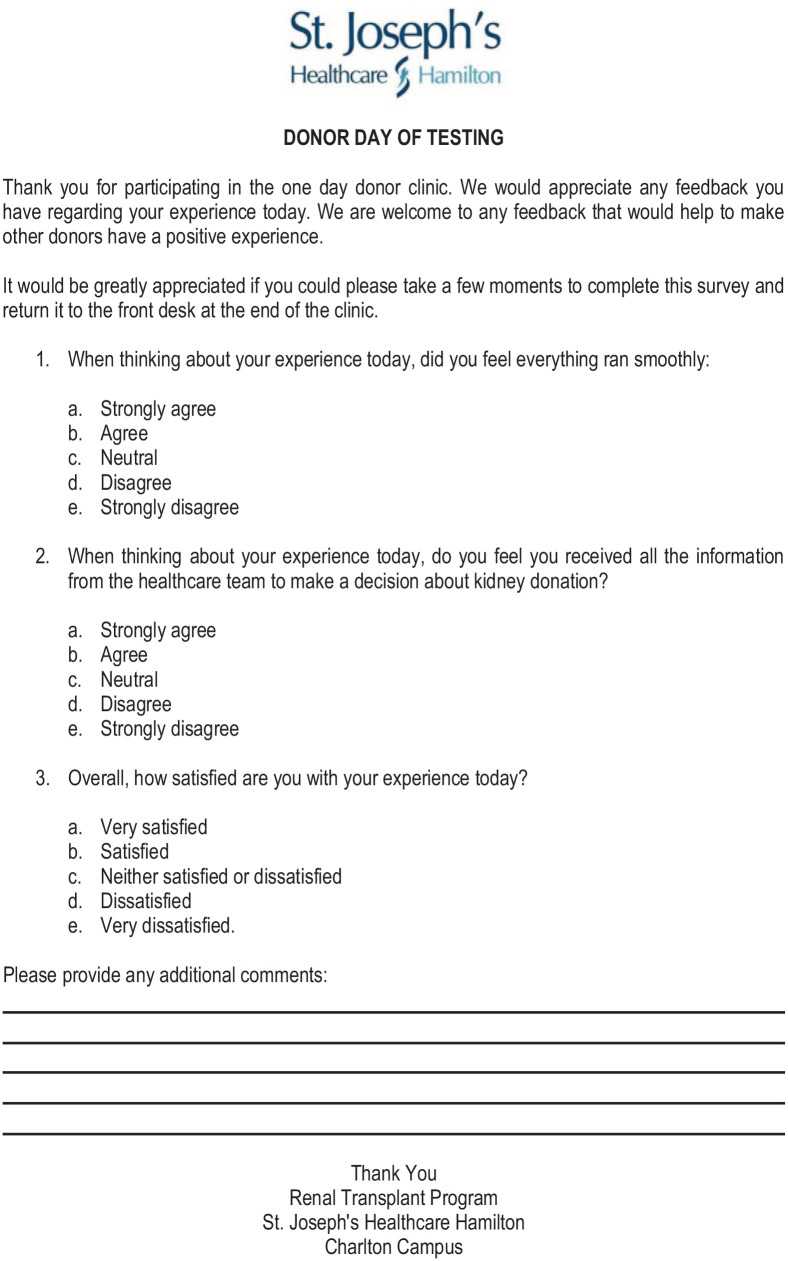
One-day donor clinic survey.

## Appendix

**Appendix A.** List of Required Testing for Kidney Donation.

Test type	Test name
Medical history	Health questionnaire, cancer screening as per Ontario guidelines (https://www.cancercareontario.ca/en/cancer-care-ontario/programs/screening-programs)
General laboratory screening	ABO blood group determination, human leukocyte antigen typing, electrolytes, bicarbonate calcium, phosphate, urea, creatinine, fasting glucose, albumin, total protein, HgbA1C, bilirubin, lipid panel, complete blood count, international normalized ratio, partial thromboplastin time, urinalysis and microscopy, urine albumin-to-creatinine ratio, urine culture
Cardiac assessment	Electrocardiogram in all donor candidates Echocardiogram and exercise stress test for all donor candidates >50 years old, or candidates between 40 and 50 with any of the following: history of possible cardiac event in past or current ischemic symptoms, risk factors for coronary artery disease such as longstanding smoking, strong family history of coronary artery disease, obesity, or hypertension
Infectious disease and virology testing	Cytomegalovirus IgG, Human T-lymphotrophic virus1 and HTLV2, Ebstein-barr virus IgG, syphilis serology, hepatitis C antibody, Human immunodeficiency virus serology, Varicella zoster virus antibody, hepatitis B Core antibody, hepatitis B surface antigen, hepatitis B surface antibody, tuberculosis skin test or equivalent in high-risk donor candidates Complete if clinically significant: hepatitis B DNA if hepatitis B core antibody or surface antigen positive, hepatitis C RNA if hep C positive antibody positive
Imaging tests	Chest x-ray, nuclear glomerular filtration rate scan, computerized tomography angiogram of the abdomen
Consultations/visit	Social work, donor coordinator, nephrologist, transplant surgeon Psychologist consultation for donor candidates who are anonymous (directed or non-directed), solicited through social media, or have a history of mental illness
